# Indium Nitride Nanowires: Low Redox Potential Anodes for Lithium‐Ion Batteries

**DOI:** 10.1002/advs.202310166

**Published:** 2024-03-27

**Authors:** Tianqi Guo, Yurong Zhou, Zhongchang Wang, Joao Cunha, Cristiana Alves, Paulo Ferreira, Zhaohui Hou, Hong Yin

**Affiliations:** ^1^ International Iberian Nanotechnology Laboratory (INL) Braga 4715‐330 Portugal; ^2^ School of Chemistry Beihang University Beijing 100191 China; ^3^ Mechanical Engineering Department and IDMEC Instituto Superior Técnico University of Lisbon Lisbon 1049‐001 Portugal; ^4^ Materials Science and Engineering Program University of Texas at Austin Austin TX 78712 USA; ^5^ Key Laboratory of Hunan Province for Advanced Carbon‐based Functional Materials School of Chemistry and Chemical Engineering Hunan Institute of Science and Technology Yueyang 414006 China

**Keywords:** electronic conductivity, high‐energy density, indium nitride nanowire, lithium‐ion batteries, low redox potential

## Abstract

Advanced lithium‐ion batteries (LIBs) are crucial to portable devices and electric vehicles. However, it is still challenging to further develop the current anodic materials such as graphite due to the intrinsic limited capacity and sluggish Li‐ion diffusion. Indium nitride (InN), which is a new type of anodic material with low redox potential (<0.7 V vs Li/Li^+^) and narrow bandgap (0.69 eV), may serve as a new high‐energy density anode material for LIBs. Here, the growth of 1D single crystalline InN nanowires is reported on Au‐decorated carbon fibers (InN/Au‐CFs) via chemical vapor deposition, possessing a high aspect ratio of 400. The binder‐free Au‐CFs with high conductivity can provide abundant sites and enhance binding force for the dense growth of InN nanowires, displaying shortened Li ion diffusion paths, high structural stability, and fast Li^+^ kinetics. The InN/Au‐CFs can offer stable and high‐rate Li delithiation/lithiation without Li deposition, and achieve a remarkable capacity of 632.5 mAh g^−1^ at 0.1 A g^−1^ after 450 cycles and 416 mAh g^−1^ at a high rate of 30 A g^−1^. The InN nanowires as battery anodes shall hold substantial promise for fulfilling superior long‐term cycling performance and high‐rate capability for advanced LIBs.

## Graphical abstract

In this study, robust InN nanowires are introduced on carbon fibers (InN/Au‐CFs) as anode for high‐performance lithium‐ion batteries (LIBs), demonstrating impressive performance with capacities of 416 mAh g^–1^ at a high rate of 30 A g^–1^. These results demonstrate the potential of the InN/Au‐CFs anode and its suitability for high‐rate LIBs.

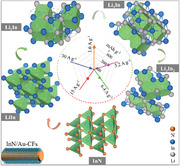

## 1 Introduction

Lithium‐ion batteries (LIBs) have emerged as the main energy source for a wide range of portable electronic devices and electric vehicles (EVs) due to their rechargeable nature and high capacity.^[^ ^1^ ^]^ With the advance of technology, the industry is increasingly seeking higher energy‐density battery materials and solutions to solve the extremely fast charging issues.^[^ ^2^ ^]^ Currently, the prevailing approach to boost the energy density of LIBs involves the increase of mass of the electrode loading by, e.g., using high‐specific capacity electrode materials and expanding the working voltage window.^[^ ^3^ ^]^ Graphite stands as the most commonly utilized anode material due to its low cost, ease of preparation, and low lithium‐ion intercalation voltage.^[^ ^4^ ^]^ Nevertheless, its limited capacity and undesirable interfacial lithium dendrite growth under high current conditions pose challenges in achieving optimal fast‐charging performance.^[^ ^5^ ^]^ Furthermore, the low density of graphite necessitates a substantial increase in thickness when being employed in high‐energy‐density (HED) cells, impeding Li‐ion diffusion.^[^ ^6^ ^]^ Consequently, the development of novel active materials with both high capacities and fast Li‐ion diffusion becomes crucial in realizing HED‐LIBs.

Indium nitride (InN), a well‐known semiconductor material, has been widely used for various applications, such as high‐frequency terahertz communications and solar energy devices.^[^ ^7^ ^]^ As a new anodic material for LIBs, InN has relatively low redox potential (< 0.7 V vs Li^+^/Li) and a high theoretical specific capacity of over 1200 mAh g^−1^, over three times that of conventional graphite anodes (∼372 mAh g^−1^).^[^ ^8^ ^]^ Moreover, InN has a narrow bandgap (0.69 eV) with good electron transfer properties.^[^ ^9^ ^]^ Therefore, InN can show fasting charging without Li dendrite due to the inhibited Li deposition, which could be applicable for next‐generation HED‐LIBs. However, its inherent volume change in the conversion and alloying/dealloying process with Li‐ions may cause pulverization of InN particles, collapse of electrode integrity, destruction of solid electrolyte interphase (SEI), and consumption of active Li‐ions.^[^ ^10^ ^]^ These issues would downgrade Li‐storage performance, greatly hindering the commercial application of InN anodes.^[^ ^11^ ^]^

To overcome these issues, various approaches have been proposed, such as the use of microstructured NiSe_2_/Ni^[^ ^12^ ^]^ and ZnO/CC^[^ ^13^ ^]^ materials, VN nanowires^[^ ^14^ ^]^ Fe_3_O_4_ nanoplates,^[^ ^15^ ^]^ Fe_2.8_Sn_0.2_O_4_@C composites^[^ ^16^ ^]^ and Si/C spheres,^[^ ^17^ ^]^ aiming to alleviate volume expansion and improve ion diffusion. Among these approaches, 1D nanostructures, e.g., nanowires, show superior physicochemical properties in virtue of the enhancement of Li‐storage and diffusion. First, 1D nanostructures can accommodate strain along specific directions during the delithiation/lithiation process.^[^ ^18^ ^]^ Second, the spacing between 1D nanostructures is large enough to provide a buffer during the volumetric structural changes of the active materials.^[^ ^19^ ^]^ Third, 1D nanostructures are beneficial to Li‐ion diffusion across the transversal direction due to the large aspect ratio, giving rise to promising rate performance.^[^ ^20^ ^]^ Moreover, 1D nanostructures can act as a self‐supporting electrode without additional non‐active components (e.g., binders), which greatly reduces the weight of electrode.^[^ ^21^ ^]^

Electrospinning, solvothermal synthesis, and chemical vapor deposition (CVD) are often effective techniques for the preparation of 1D nanowires. In particular, the CVD, known for its simplicity, short experimental cycles, and production of highly crystalline products, has found wide application in the preparation of 1D semiconductor nanostructures.^[^ ^22^ ^]^ During this process, gold or silver is often used as a catalyst or growth site, which is crucial for semiconductor materials that require high structural precision. These noble metals can effectively control the crystallography and growth direction of the grown materials. Furthermore, since gold and silver are excellent electric conductors, they are also employed to enhance the conductivity of electrode materials, thereby improving the electrochemical performance of the electrodes.^[^ ^23^ ^]^ Through this approach, it is possible to enhance the overall performance of electrochemical devices while maintaining material structural precision.

Here, we propose 1D single‐crystalline InN nanowires grown on Au‐decorated carbon fibers (InN/Au‐CFs) as a new anode material for LIBs. We systematically investigate the structural properties and Li‐storage performance of the InN/Au‐CFs composite. Further density functional theory (DFT) calculations are employed to assess the density of states and adatom adsorption of the InN nanowires. We find that ion diffusion, reactivity, and electrode stability are improved. Notably, the InN/Au‐CFs anode exhibits an impressive capacity, delivering 632.5 mAh g^−1^ at a rate of 0.1 A g^−1^ after 450 cycles and 416 mAh g^−1^ at a rate of 30 A g^−1^. The full cells that are paired with commercial LiNi_0.8_Co_0.15_Zn_0.05_O_2_ cathode deliver long‐term cycling stability, suggesting that the InN nanowires can hold promise as a prospective material for high‐energy‐density and high‐power‐density LIBs.

## 2 Results and Discussion

### 2.1 Structural Characterization

**Figure 1:**
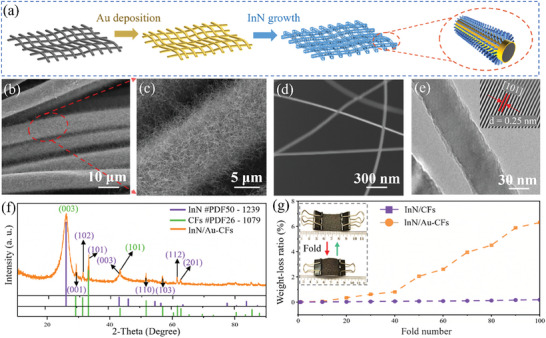
a) Schematic illustrating the preparation of InN/Au‐CFs composite. b–d) SEM images of the InN/Au‐CFs composite at different magnifications. e) TEM image of InN nanowires (insertion: lattice spacing of 0.25 nm of the (101) planes). f) XRD pattern illustrating well‐indexed peak positions corresponding to the InN/Au‐CFs composite g) Weight‐loss ratios at different fold times, highlighting the enhanced stability of the InN/Au‐CFs composite compared to the InN/CFs composite.

**Figure** **1** presents the overall synthesis route and structural characterization. The process starts with the activation of carbon fibers (CFs in Figure 1a) in an HNO_3_‐H_2_SO_4_ solution to enhance their hydrophilicity. After the acid treatment, Au‐decorated CFs (Au‐CFs) are prepared by magnetron sputtering. The contact angle decreases to 41.7° (Figure S1, Supporting Information), indicating enhanced hydrophilicity compared to the pristine CFs. Scanning electron microscopy (SEM) images (Figure S2, Supporting Information) reveal that the CFs have a mean diameter of ≈10 µm and Au decoration causes no change in morphology. In addition, SEM mapping of Au‐CFs (Figure S3, Supporting Information) and TEM imaging also reveal that the size of Au nanoparticles is ≈8 nm (Figure S4, Supporting Information). These gold nanoparticles exhibit favorable hydrophilicity and provide numerous growth sites for InN nanowires.^[^ ^24^ ^]^

InN nanowires are further grown on the surface of Au‐CFs (Figure 1b) by the CVD. InN nanowires are uniformly and densely distributed on the surface of Au‐CFs (denoted as InN/Au‐CFs). A high‐magnification SEM image (Figure 1c,d) and distribution of diameter in Figure S5 (Supporting Information) reveal that InN nanowires possess an average diameter of 50 nm. The length of InN nanowire is ≈20 µm, leading to a high aspect ratio of 400, which contributes to the shortening of Li‐ion diffusion paths and the excellent flexibility.^[^ ^25^ ^]^ The structure of these InN nanowires can be further observed by TEM (Figure 1e). The (101) planes of hexagonal InN are identified with the corresponding d‐spacing of 0.25 nm.^[^ ^26^ ^]^ Moreover, the InN/Au‐CFs composite demonstrates favorable wettability with Ethylene Carbonate (EC)/Propylene Carbonate (PC) (Figure S6, Supporting Information). These characteristics are expected to enhance electrode stability and cycling performance of the InN/Au‐CFs composite. In contrast, Figure S7 (Supporting Information) displays the morphology of InN nanowires on CFs with respect to gold deposition times. It suggests a non‐uniform and less dense distribution of InN nanowires, which may lead to poor cycling performance. It is worth noting that at high temperature and vacuum condition, gold particles are transitioned into a highly adsorbent state. Ammonia molecules accumulate on the surfaces of these gold particles, while indium vapor is also adsorbed on the surfaces of gold particles and reacts with ammonia to form InN nanowires. Additionally, the presence of gold particles declines the activation energy of the reaction, facilitating material growth.

The crystal structure of the InN/Au‐CFs composite was examined using X‐ray diffraction (XRD), as shown in Figure 1f, which displays well‐indexed peak positions corresponding to InN (JCPDS card No. 50–1239) and carbon fibers (JCPDS card No. 26–1079). The characteristic peaks for hexagonal InN with a P63mc space group, namely, the (001), (102), and (101) planes at 2*θ* = 29.5°, 31.5°, and 33°, respectively, as well as the (003) at 2*θ* = 26° and (101) at 2*θ* = 43° planes corresponding to the carbon fiber substrate, are observed. The XRD pattern of pure carbon fibers is shown in Figure S8 (Supporting Information). It is worthy of mentioning that the XRD pattern does not exhibit diffraction peaks associated with Au nanoparticles due to their low content and the fact that they are uniformly coated by InN nanowires. To further elucidate componential properties of the InN/Au‐CFs, X‐ray photoelectron spectroscopy (XPS) was employed. In Figure S9a,b (Supporting Information), distinct peaks related to In 3*d* and N 1*s* are observed. Specifically, the XPS spectra reveal the electron‐binding energy of In 3*d*_3/2_ at 453.2 eV and In 3*d*_5/2_ at 445.2 eV, indicating the existence of In─N bond.^[^ ^27^ ^]^ The binding energies of N 1*s* are found at 401.2 and 399.3 eV, confirming the presence of N (III) anions in InN. Raman spectroscopy (Figure S9c, Supporting Information) further supports the identification of InN and carbon fiber components in the sample. The specific adsorption peaks are observed, which correspond to InN and the D and G bands located at 1299.7 and 1585 cm^−1^, respectively, as well as carbon fibers, consistent with the previous report.^[^ ^28^ ^]^ These results consolidate the well‐defined crystallinity and composition of the synthesized InN/Au‐CFs.

The content of InN nanowires within the carbon fibers was determined by the thermogravimetric analysis coupled with differential scanning calorimetry (TGA‐DSC) (Figure S9d, Supporting Information). Based on the weight loss curve, the mass ratio of InN nanowires is estimated to be ≈8.2 wt.%.^[^ ^29^ ^]^ To assess the stability of InN nanowires on carbon fibers, fold tests were conducted and weight loss ratios at different fold times were measured (Figure 1g). Remarkably, the mass of InN/Au‐CFs exhibits minimal fluctuation with a weight‐loss ratio of only 0.19% after 100‐fold times. In contrast, the InN/CFs show a significant weight loss, reaching 6.31% after 100‐fold iterations. These findings suggest that the presence of Au nanoparticles enhances the binding force between Au and InN and the stability of InN nanowires on carbon fibers.

### 2.2 Li‐Storage Properties of the InN/Au‐CFs Anode

**Figure 2:**
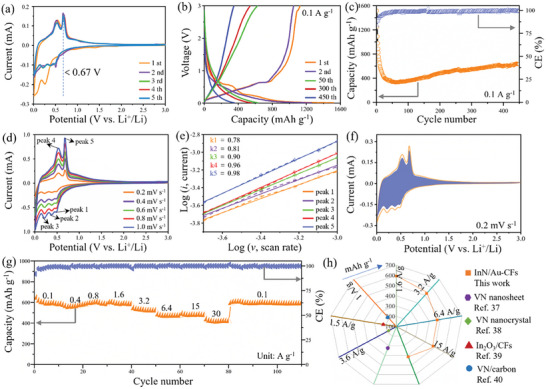
a) CV curves with a voltage range of 0.01–3.0 V at a scan rate of 0.1 mV s^−1^. b) Discharge/charge profiles at 0.1 A g^−1^. c) Cycling performance of the InN/Au‐CFs anode at 0.1 A g^−1^. d) CV profiles at various scan rates from 0.2 to 1.0 mV s^−1^. e) Relationship between peak current *i* and scan rate v in a log (*i*) versus log (*v*) representation, with respective fits. f) Contribution of pseudocapacitive capacity at 0.2 mV s^−1^. g) Rate performance at various current rates from 0.1 to 30 A g^−1^. h) Comparison of the rate performance of the nitride/indium‐based Li‐ion anodes reported in the literature.

The electrochemical properties of InN/Au‐CFs as an anode material for LIBs are evaluated in multiple CR2032‐type coin cells with Li foil as both counter and reference electrodes. The initial five cyclic voltammetry (CV) curves of the InN/Au‐CFs anode were measured at a scan rate of 0.1 mV s^−1^ (vs Li^+^/Li) from 0.01 to 3 V. As shown in **Figure** **2**a, an inconspicuous reduction peak is identified at 0.52 V, which can be attributed to the conversion and alloying processes of InN into Li_3_N and LiIn, respectively.^[^ ^30^ ^]^ The peak is shifted to 0.37 V in the following cycles. A sharp peak at 0.26 V can be ascribed to the formation of the solid electrolyte interphase (SEI), consistent with the previous reports.^[^ ^31^ ^]^ Subsequently, this peak evolves into two peaks located at 0.37 and 0.21 V during the following cycles due to the formation of Li_2_In and Li_3_In, respectively. A sharp peak at 0.53 V can be associated with the formation of Li_3_In_2_ during the corresponding anodic scan. The next anodic peak at 0.68 V can correspond to the formation of InN. The oxidation and reduction peaks in the CV curves reveal an excellent overlap during the subsequent cathodic and anodic scans, suggesting good reversibility and stability of the InN/Au‐CFs anode. All redox peaks below 0.7 V indicate that the InN/Au‐CFs anode could provide a wide voltage window, holding great promise for the HED‐LIBs.^[^ ^32^ ^]^

This behavior is also shown from the charge/discharge profiles (Figure 2b) for the 1st, 2nd, 50th, 300th, and 450th cycles at a current density of 0.1 A g^−1^. The discharge plateaus from 0.25 to 0.5 V and the charge plateaus from 0.5 to 1.0 V coincide with the oxidation and reduction peaks of CV curves, respectively. In the first cycle, the InN/Au‐CFs anode displays discharge and charge capacities of 1517 and 1265 mAh g^−1^ at 0.1 A g^−1^, respectively, leading to an initial coulombic efficiency (CE) of 83.4%. The irreversible capacity loss can be ascribed to the formation of SEI film and irreversible side reactions with the electrolyte. In addition, it is worth noting that the Au nanoparticles might decrease CE of the InN/Au‐CFs anode.^[^ ^33^ ^]^ In comparison, Figure S10 (Supporting Information) shows the charge/discharge profiles of the InN/CFs anode, suggesting much lower capacities and poorer stability than those of the InN/Au‐CFs anode. The cycling stability of the InN/Au‐CFs anode is illustrated in Figure 2c, where remains almost unaltered after 50 cycles. In particular, the specific capacity of the InN/Au‐CFs anode is stabilized ≈632.5 mAh g^−1^ at 0.1 A g^−1^ over 450 cycles, and the CE is approximately constant at 99% since the 10th cycle. In contrast, the InN‐CFs anode only delivers a capacity of 167.5 mAh g^−1^ after 450 cycles at 0.1 mA g^−1^ (Figure S11, Supporting Information).

Furthermore, during the analysis of electrode structures after the extended cycling, we observed through the SEM and TEM images that even after long‐term cycling, InN nanowires maintain their original nanowire structure (Figure S12, Supporting Information). This suggests that InN nanowires exhibit excellent structural stability during cycling. Hence, the InN/Au‐CFs anode exhibits higher specific capacities and more stable cycle performance than the InN‐CFs anode. This can be attributed to the structural advantages of InN/Au‐CFs anode in the following aspects. First, the Au nanoparticles effectively enhance interfacial properties between carbon fibers and InN nanowires, providing excellent electronic transport channels. Second, the Au nanoparticles offer growth sites for InN nanowires, which significantly improves adhesion between the CFs and InN nanowires and prevents peeling‐off of active materials from the substrate at long‐term cycling.

To better evaluate the rate performance of the InN/Au‐CFs anode and determine the maximum achievable rate current, we first investigated charge storage mechanism of the as‐prepared electrode by CV at various scan rates from 0.2 to 1.0 mV s^−1^. The obtained CV curves suggest that the capacitive characteristics and diffusion‐limited redox reactions dominate the charge/discharge processes. As shown in Figure 2d, one pair of clear cathodic peaks at 0.48, 0.37, and 0.21 V, and two anodic peaks at 0.53 and 0.68 V are noticeably observed, which recur intensively in the following scans. The peak evolution over several scans can be ascribed to the non‐Faradaic or Faradaic processes (Appendix S1, Supporting Information). Figure 2e exhibits the linearity between log (*i*) and log (*v*) plots, where *i* and *v* are the current and scan rate, respectively, at different peak potentials. The slope of the log (*i*) versus log (*v*) curves is 0.78, 0.81, 0.90, 0.96, and 0.98 for the different CV peaks, indicating the electrochemical properties are predominantly controlled by pseudocapacitive processes within the InN/Au‐CFs anode, which results in fast Li‐ion diffusion and excellent rate capability.

Figure 2f shows the pseudocapacitive contribution ratio of the InN/Au‐CFs anode at 0.2 mV s^−1^. The pseudocapacitive contribution ratios at different scan rates are shown in Figure S13 (Supporting Information). The calculated pseudocapacitive contribution ratios are 88.30%, 91.09%, 92.27%, 94.97%, and 97.95% at 0.2, 0.4, 0.6, 0.8, and 1 mV s^−1^, respectively, confirming that the pseudocapacitive process dominates the Li‐storage capacity. These pseudocapacitive Li‐ion storage processes contribute to the excellent rate performance of the InN/Au‐CFs anode. For the rate performance, the cell was first pre‐cycled for 50 cycles. The rate performance test uncovers that the InN/Au‐CFs anode can deliver high reversible capacities of 584, 560, 594, 584, 525, 474, 478, and 416 mAh g^−1^ at 0.1, 0.4, 0.8, 1.6, 3.2, 6.4, 15 and 30 A g^−1^, respectively (Figure 2h). When the current density returns to 0.1 A g^−1^ after 100 cycles, the specific capacity is 597 mAh g^−1^. For comparison, the InN‐CFs anode shows lower rate capacities than InN/Au‐CFs anode, consistent with the cycling test results (Figure S14, Supporting Information). We also compare our results with other nitride/indium‐based lithium‐ion anodes reported in literatures, such as VN and In_2_O_3_ (Figure 2h). All the reported maximum rate currents are below 3.2 A g^−1^, much lower than that in our work, which indicates superior rate performance of InN/Au‐CFs anode in comparison with that of the VN nanosheet/nanocrystalline/carbon‐coated fibers and In_2_O_3_‐carbon fibers.^[^ ^34^ ^]^

### 2.3 Li‐Storage Mechanism of the InN/Au‐CFs Anode

**Figure 3:**
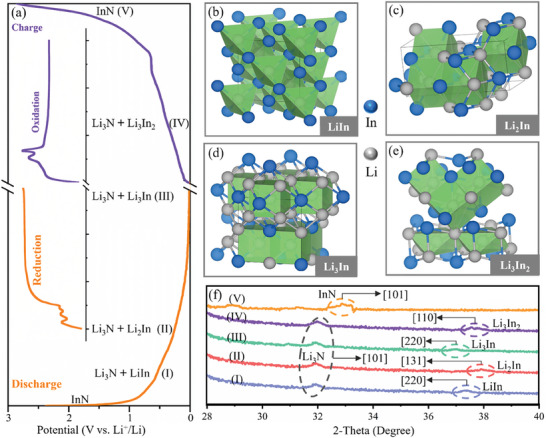
a) The charge/discharge profiles of the InN/Au‐CFs anode at 0.1 A g^−1^, the insert is the CV curves. b–e) The crystal structures of the cubic LiIn with a space group of Fd3̅m1, orthorhombic Li_2_In with a space group of Cmcm, cubic Li_3_In with a space group of Pm3̅m and trigonal Li_3_In_2_ with a space group of R3̅m, respectively. f) In situ XRD pattern evolution of the InN/Au‐CFs anode during the second charge/discharge process at 0.01‐3.0 V.

To further clarify the Li‐storage mechanism of the InN/Au‐CFs anode, in situ, XRD tests were conducted on an *operando* cell. **Figure** **3**a shows the charge/discharge curves at 0.1 A g^−1^ during the in situ XRD test. The crystal structures of the main intermediate phases are schematically shown in Figure 3b–e, including the cubic LiIn (space group Fd3̅m1), orthorhombic Li_2_In (space group Cmcm), cubic Li_3_In (space group Pm3̅m) and trigonal Li_3_In_2_ (space group R3̅m). In the discharge process, the Li─In bonds gradually become stronger, transforming into a borane structure of the same group.^[^ ^35^ ^]^ The crystal structure of Li_3_N is also shown in Figure S15 (Supporting Information). In the charge process, the In atoms rebuild the bonds and the Li atoms redistribute in the cell.^[^ ^36^ ^]^

We also performed XRD analysis at different lithiation/delithiation stages of the discharge/charge profiles. Figure 3f displays the corresponding XRD patterns at various stages. A five‐stage reaction mechanism is proposed for the InN/Au‐CFs anode. In stage I (0.48 V, lithiation), the characteristic peaks of the Li_3_N with (220) plane and LiIn with (015) plane are observed, which suggests that the sample is composed of Li_3_N and LiIn. It should be noted that the Li_3_N phase is present within the entire lithiation/delithiation process. In stage II (0.37 V, lithiation), the patterns can be assigned to the intermediate phase of Li_2_In.^[^ ^37^ ^]^ Subsequently, the Li_2_In phase is transformed into Li_3_In in stage III at 0.21 V. During the charging process, the characteristic peaks of the Li_3_In_2_ are identified at 0.53 V in stage IV. In stage V, the Li_3_In_2_ reacts with Li_3_N, forming the hexagonal InN whose characteristic peaks are located at 35°, similar to the original diffraction peaks (Figure 2f). The overall lithiation/delithiation reaction equations of the InN/Au‐CFs anode can be expressed as follows.

Lithiation:

(1) $$\left(I\right)\mathrm{InN}+4Li^{+}+4e^{-}\to \mathrm{LiIn}+Li_{3}N\;0.48\;\;V$$

(2) $$\left(\mathrm{II}\right)\;\mathrm{LiIn}+Li_{3}N+Li^{+}+e^{-\;}\to Li_{2}\mathrm{In}+Li_{3}N\;0.37\;V$$

(3) $$\left(\mathrm{III}\right)Li_{2}\mathrm{In}+Li_{3}N+Li^{+}+e^{-}\to Li_{3}\mathrm{In}+Li_{3}N\;0.21\;V$$

Delithiation:

(4) $$\left(\mathrm{IV}\right)Li_{3}\mathrm{In}+Li_{3}N-1.5Li^{+}\to 0.5Li_{3}In_{2}+Li_{3}N+1.5e^{-}\;0.53\;V$$

(5) $$\left(V\right)\;0.5Li_{3}In_{2}+Li_{3}N-4.5Li^{+}\to \mathrm{InN}+4.5e^{-}\;0.68\;\;V$$

**Figure 4:**
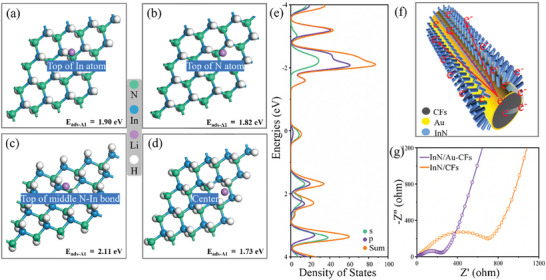
a–d) The selected configurations and adsorption energies of Li‐ion adsorbed on the InN nanowire (101) surface, respectively. e) The density of states of InN nanowire on the surface of Au nanoparticles. h) The illustration of electronic transportation. g) The Nyquist plots of the impedance spectra.

To understand structure evolution during lithiation/delithiation, the adsorption energy of Li‐ion on InN with (101) plane was calculated given that this is the growth direction of the InN nanowire. We considered the optimized configurations of four possible sites and corresponding adsorption energies, which are located at the In atom top (**Figure** **4**a), the N atom top (Figure 4b), the middle of the N─In bond top (Figure 4c), and the center (Figure 4d). The four adsorption energies are similar and the Li‐ion adsorption induces insignificant geometric deformation or lattice expansion, implying stable volume during lithiation/delithiation and promising potential application in LIBs.^[^ ^38^ ^]^ Furthermore, to study the electrical conductivity of the InN/Au‐CFs composite, we also conducted the density of states (DOS) analyses (Figure 4e). The valence band passes through the Fermi level, suggesting conducting nature. This indicates that the Au‐modified layer could adjust the InN semiconductor properties. As such, electrons experience fast transport from the CFs substrate to the InN nanowires through the Au‐modified layer (Figure 4f).

Electrochemical impedance spectroscopy (EIS) further confirms the conductivity of the InN/Au‐CFs anode and clarifies Li‐ions diffusion mechanism in the discharge/charge process. From the EIS fitting to the equivalent circuit in Depiction S2, the charge‐transfer resistance (R_ct_) is evaluated to be 120 Ω for InN/Au‐CFs and 380 Ω for InN‐CFs anodes (Figure 4g). Consequently, the InN/Au‐CFs anode shows higher electronic conductivity and ion diffusion rate than the InN/CFs anode. Additionally, the Li‐ion diffusion coefficient (*D*), which directly influences the rate performance of anode materials, can be obtained from the EIS fitting.^[^ ^39^ ^]^ Particularly, *D* is directly related to the Warburg factor (*σ*) (see Appendix S2, Supporting Information) as *σ* ^2^ is inversely proportional to *D*,^[^ ^40^ ^]^ From Figure S16 (Supporting Information), the *σ* are calculated to be 119.8 and 445.6 Ω s^−1/2^ for the InN/Au‐CFs and InN‐CFs anodes, respectively, implying a larger diffusion coefficient for the InN/Au‐CFs.

### 2.4 Full cell performance of the InN/Au‐CFs Anode

**Figure 5:**
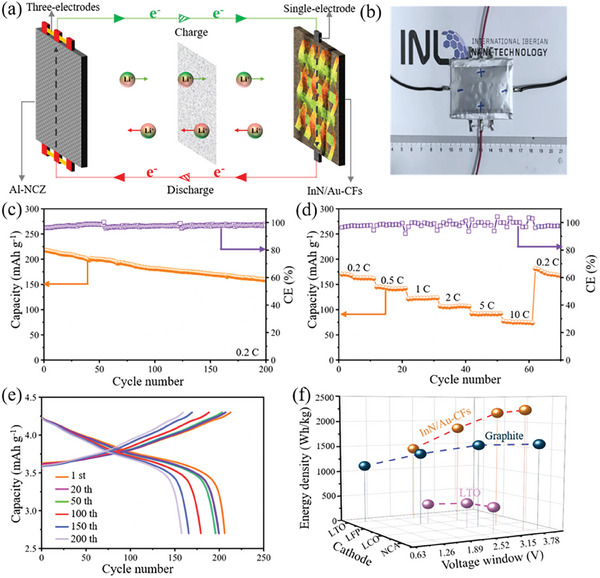
a) schematic illustration of the full cell including Al‐NCZ as the cathode and InN/Au‐CFs as the anode. b) A photograph of the two‐terminal electrode battery. c) The cycling performance of the Al‐NCZ||InN/Au‐CFs full cell at 0.2 C. d) The rate performance of the Al‐NCZ||InN/Au‐CFs full cell. e) Discharge/charge profiles at different cycling number at 0.2 C. f) Energy density of different combinations of anodes and cathodes for full cells.

The electrochemical performance of the InN/Au‐CFs was evaluated in a full cell configuration using the Al_2_O_3_‐coated LiNi_0.8_Co_0.15_Zn_0.05_O_2_ (Al‐NCZ) as cathode material. The selection of Al‐NCZ as the cathode material is based on its superior Li‐storage performance in half cells (theoretical capacity 279 mAh g^−1^, 1 C = 0.279 A g^–1^),^[^ ^41^ ^]^ making it a promising candidate for LIBs due to its high voltage plateaus, favorable performance, and cost‐effectiveness. The preparation details of the Al‐NCZ cathode are provided in Appendix S3 (Supporting Information). **Figure** **5**a illustrates the assembly of the Al‐NCZ||InN/Au‐CFs full cell. The InN/Au‐CFs anode reaction exchanges 6 electrons in total, while the Al‐NCZ electrode reaction exchanges one Li‐ion. The overall reaction of the full cell can then be expressed as follows:

(6) $$\begin{array}{ccc} & & 6\mathrm{LiN}i_{0.8}Co_{0.15}Zn_{0.05}O_{2}+x\mathrm{InN}\\ & & \;↔6Li_{1-x}Ni_{0.8}Co_{0.15}Zn_{0.05}O_{2}+xLi_{3}N+xLi_{3}\mathrm{In} \end{array}$$

To achieve stable high‐rate performance in the full‐cell configuration, a two‐terminal electrode design was implemented, as depicted in Figure 5b. This design helps mitigate metal electrode fatigue at high rates and improves the rate performance of conventional electrode materials. The cyclic voltammetry (CV) and the charge/discharge profiles of the full cell are presented in Figure S17 (Supporting Information), consistent with the previous reports.^[^ ^42^ ^]^ The full cell shows a capacity of 159.5 mAh g^−1^ at 0.2 C after 200 cycles based on the weight of Al‐NCZ (Figure 5c), indicating favorable long‐term cycling performance. The corresponding anode capacity is measured as 565 mAh g^−1^. The rate performance of the full cell with 50 pre‐cycles at 0.2 C was evaluated at different current rates, as shown in Figure 5d. The full cell yields a capacity of 70.5 mAh g^−1^ at 10 C, which recovers to 172.3 mAh g^−1^ when the current is reduced back to 0.2 C. The discharge “plateau” ranges from 3.30 to 3.75 V, while the charge “plateau” spans from 3.6 to 4.2 V, in agreement with the oxidation and reduction peaks observed in the CV curves (Figure 5e).

The energy density of the InN/Au‐CFs anode was systematically evaluated and compared with commercial anodes such as graphite and lithium titanate (LTO), as depicted in Figure 5f. Additionally, the reference cathodes including the LiFePO_4_ (LFP), LiCoO_2_ (LCO), and LiNi*_x_* Co_y_Al_1‐_ *_x_*_‐y_O_2_ (NCA) were also considered.^[^ ^43^ ^]^ The InN/Au‐CFs anode exhibits the highest energy density owing to its high capacity and wide voltage window. In contrast, the LTO anode displays the lowest energy density due to its low capacity and narrow voltage window. Such remarkable large‐rate performance and high specific capacity of the InN/Au‐CFs anode in full cells demonstrate its great potential in the application of HED‐LIBs.

## 3 Conclusion

To solve the key challenges in applying InN for LIBs such as poor ion diffusion, limited reactivity, and structural instability, we have grown InN nanowires of high aspect ratio on Au‐decorated carbon fibers as an InN/Au‐CFs composite anode using chemical vapor deposition method and demonstrated that the InN nanowires could act as a new high‐performance anode material. The InN nanowires have a diameter of ≈50 nm, which can effectively reduce ion diffusion path. Further theoretical calculations suggest favorable reactivity, specifically along the (101) surface growth direction for the InN nanowires. The presence of Au nanoparticles is found to provide stable growth sites for the InN nanowires, enhance electronic transport, and improve electrode stability. The InN/Au‐CFs anode exhibits outstanding electrochemical performances with a capacity of 632.5 mAh g^−1^ at 0.1 A g^−1^ after 450 cycles and of 416 mA h g^−1^ at a high‐rate of 30 A g^−1^, showing excellent high‐rate charge/discharge capability. This work demonstrates the potential of applying this new InN anode for HED‐LIBs, thus contributing to the development of high‐performance LIBs through employing nitride semiconductor materials.

## 4 Experimental Section

### Preparations of InN/Au‐CFs

The InN/Au‐CFs were synthesized in a spilled horizontal furnace. First, the carbon fibers (CFs) were boiled in a mixture of concentrated nitric acid and sulfuric acid (Vol. 3:1) at 90 °C for 3 h, followed by ultra‐sonication in water and drying in an oven at 100 °C for 2 h. Next, the surface of CFs was decorated by Au nanoparticles by using electron beam evaporation in high vacuum. The treated CFs were adopted as substrates to grow InN nanowires. A piece of 10 × 10 × 1 mm indium (In) foil (99.9%, Aldrich) was placed onto a quartz boat and loaded into the middle of a quartz tube as a source. The CFs substrate was placed downstream 15 centimeters away from the In source. Under 100 sccm (standard cubic centimeters per minute) of ammonia, the furnace temperature was raised to 900 °C with a rate of 30 °C min^−1^. The InN/Au‐CFs were obtained after reaction for 1 h, and the furnace was cooled to room temperature under argon flow. The untreated CFs (without Au nanoparticles) used as a substrate for the growth of InN nanowires (InN‐CFs) were adopted as a reference sample.

### Materials Characterization

Morphology of the InN/Au‐CFs was characterized by scanning electron microscope (SEM) (NOVA 450, Thermo Scientific) and transmission electron microscope (TEM) (G2‐F30, Thermo Scientific) coupled with energy dispersive spectroscopy (EDS). The crystalline structures of the as‐prepared nanofibers were characterized by X‐ray diffraction (X‐PERT‐PRO‐MRD, PANalytical). Raman spectra were collected on a Thermo scientific FT‐Raman spectrometer (FRA 106/s) using an Nd‐line laser source with an excitation wavelength of 532 nm. Analysis of chemical composition of the NSSCs was performed with TG measurement (Diamond TGA/DSC 6300) at a heating rate of 5 °C min^−1^ in air. The chemical states of the elements in the InN/Au‐CFs were conducted by X‐ray electron spectroscopy (XPS, ESCALAB 250Xi, Thermo Scientific). The contact angles of the solvents on the carbon surface were measured by a Drop Shape Analysis Contact Angle model DSA 100 at room temperature in air.

### Electrochemical Measurements

The as‐prepared InN/Au‐CFs sample was cut into circular samples (d = 6 mm) to fabricate working electrodes without introduction of polymeric binder and conductive additives. The average weight of the active material was ≈1.5 mg cm^−2^. The cells were assembled by adopting lithium foil as the counter electrode, 1 m LiPF_6_ in a mixture of ethylene carbonate/dimethyl carbonate (EC/PC, 1:1 in volume) as the electrolyte, and Celgard3501 (Celgard, LLC Corp., USA) as the separator. The full cell with an N/P ratio of 1.05 was assembled using Al_2_O_3_‐LiNi_0.8_Co_0.15_Zn_0.05_O_2_ as cathode.^[^ ^41^ ^]^ All cells were assembled in a glove box with water/oxygen content of lower than 0.1 ppm and tested at room temperature. The galvanostatic charge‐discharge test was conducted using a LAND cycler (CT 2001, Wuhan Kingnuo Electronic Co., China). Cyclic voltammetry and electrochemical impedance spectroscopy measurements of the coin cells were carried out using an electrochemical workstation (DH7000C, Jiangsu Donghua Analysis Instruments Co. Ltd.).

### Density Functional Theory (DFT) Calculations

The DFT calculations were performed using the plane‐wave pseudopotential formalism implemented in the CASTEP package. The exchange correlation function was treated using the generalized gradient approximation (GGA) with the Perdew‐Burke‐Ernzerhof (PBE) functional. Atomic structures were optimized by using the conjugated gradient technique to directly minimize the Kohn–Sham energy functional and the pseudopotentials to describe core electrons.

## Conflict of Interest

The authors declare no conflict of interest.

## Supporting information

Supporting Information

## Data Availability

The data that support the findings of this study are available from the corresponding author upon reasonable request.
