# Supplementary material for: Indium Nitride Nanowires: Low Redox Potential Anodes for Lithium‐Ion Batteries
Source: Adv Sci (Weinh). 2024 Mar 27;11(22):2310166. doi: 10.1002/advs.202310166 (PMC11165543; doi:10.1002/advs.202310166)
Supplement: Supplementary file 1 — Supporting Information [file ADVS-11-2310166-s001.pdf]

ADVANCED SCIENCE
Open Access

# Supporting Information

for *Adv. Sci.*, DOI 10.1002/advs.202310166

Indium Nitride Nanowires: Low Redox Potential Anodes for Lithium-Ion Batteries

*Tianqi Guo, Yurong Zhou, Zhongchang Wang*, Joao Cunha, Cristiana Alves, Paulo Ferreira*, Zhaohui Hou and Hong Yin**

# Supporting Information

## Indium Nitride Nanowires: Low Redox Potential Anodes for Lithium-Ion Batteries

*Tianqi Guo[1#], Yurong Zhou[1#], Zhongchang Wang[1,5]*, Joao Cunha[1], Cristiana Alves[1], Paulo Ferreira[1,3,4]*, Zhaohui Hou[2], and Hong Yin[1,2]**

*E-mail: zhongchang.wang@inl.int (Z.W.), paulo.ferreira@inl.int (P.F.), hong.yin@inl.int (H.Y.)

[1]International Iberian Nanotechnology Laboratory (INL), Braga 4715-330, Portugal

[2]Key Laboratory of Hunan Province for Advanced Carbon-based Functional Materials, School of Chemistry and Chemical Engineering, Hunan Institute of Science and Technology, Yueyang, 414006, China

[3]Mechanical Engineering Department and IDMEC, Instituto Superior Técnico, University of Lisbon, 1049-001 Lisbon, Portugal

[4]Materials Science and Engineering Program, University of Texas at Austin, Austin, Texas, 78712, USA

[5]School of Chemistry, Beihang University, Beijing 100191, China

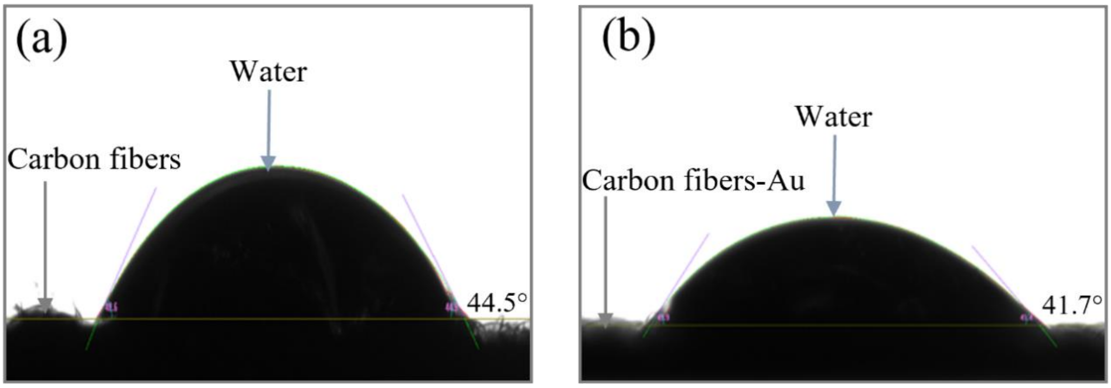


**Figure S1** (a) Contact angle measurements at the interface of carbon fibers with water solvent. (b) Contact angle measurements at the interface of Au-CFs with water solvent.

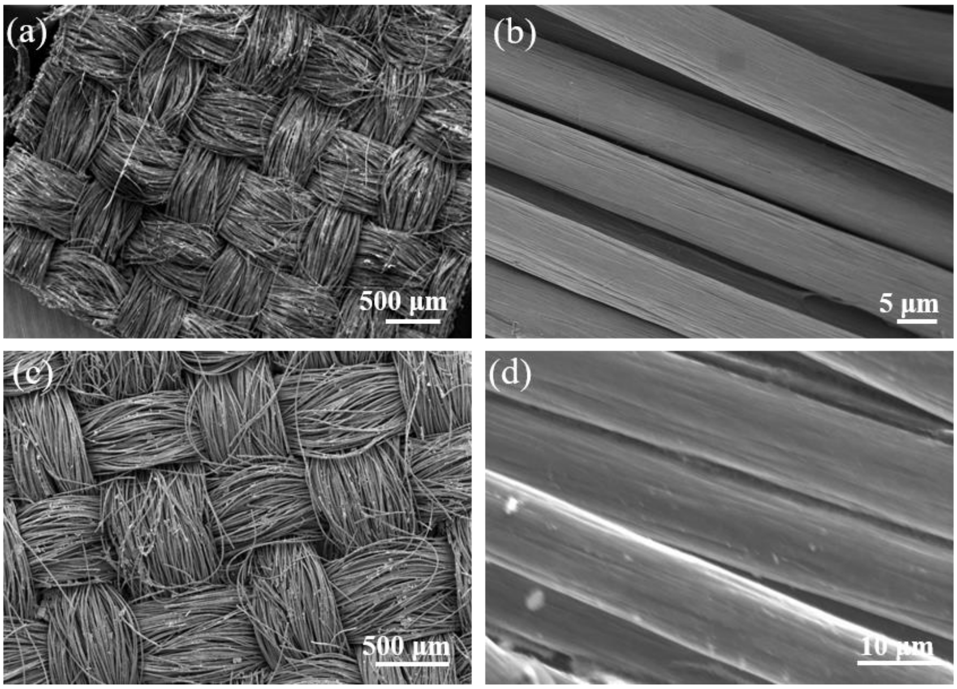


**Figure S2** SEM images of the carbon fibers at (a) low and (c) higher magnifications. SEM images of the Au-CFs at (c) low and (d) higher magnifications.

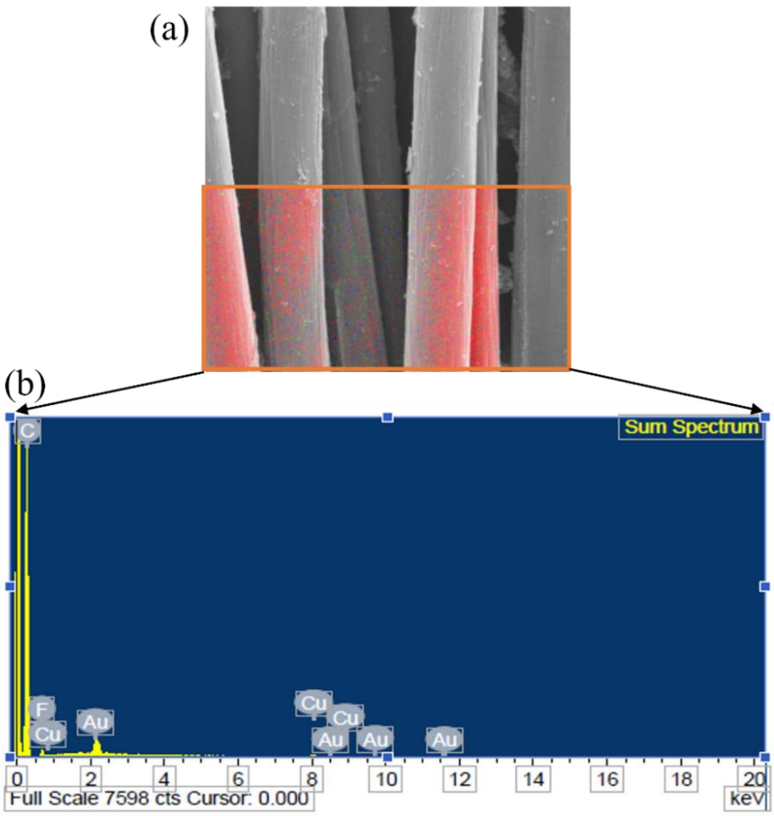


**Figure S3** (a) Mix element mapping of carbon, gold, and copper. (b) EDS spectra of Au-CFs composite.

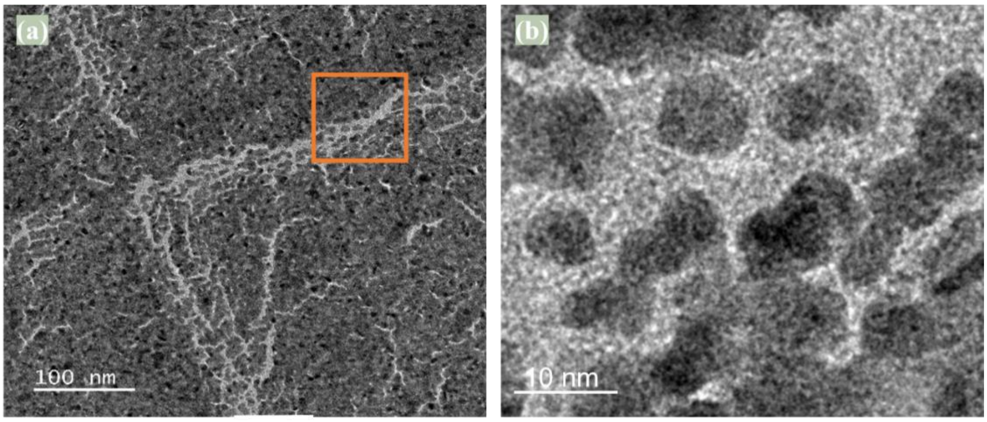


**Figure S4** (a) TEM image of gold nanoparticles. (b) Select area magnification of (a).

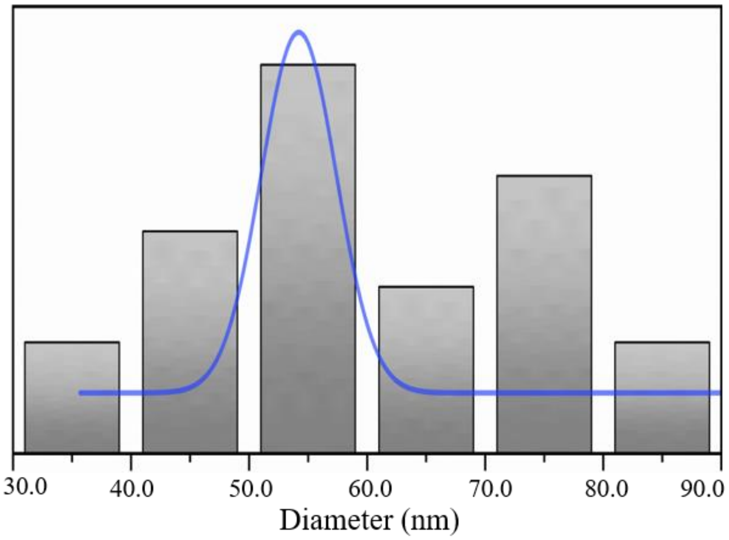


**Figure S5** The diameter distribution of the InN nanowires obtained from SEM images.

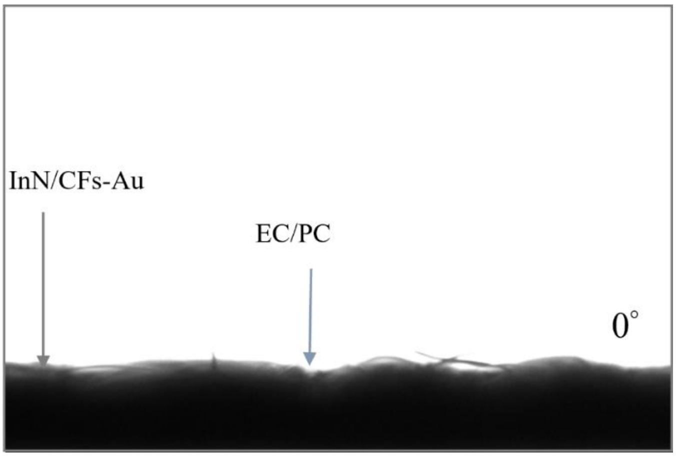


**Figure S6** Contact angle measurements at the interface of InN/Au-CFs composite with water EC/PC.

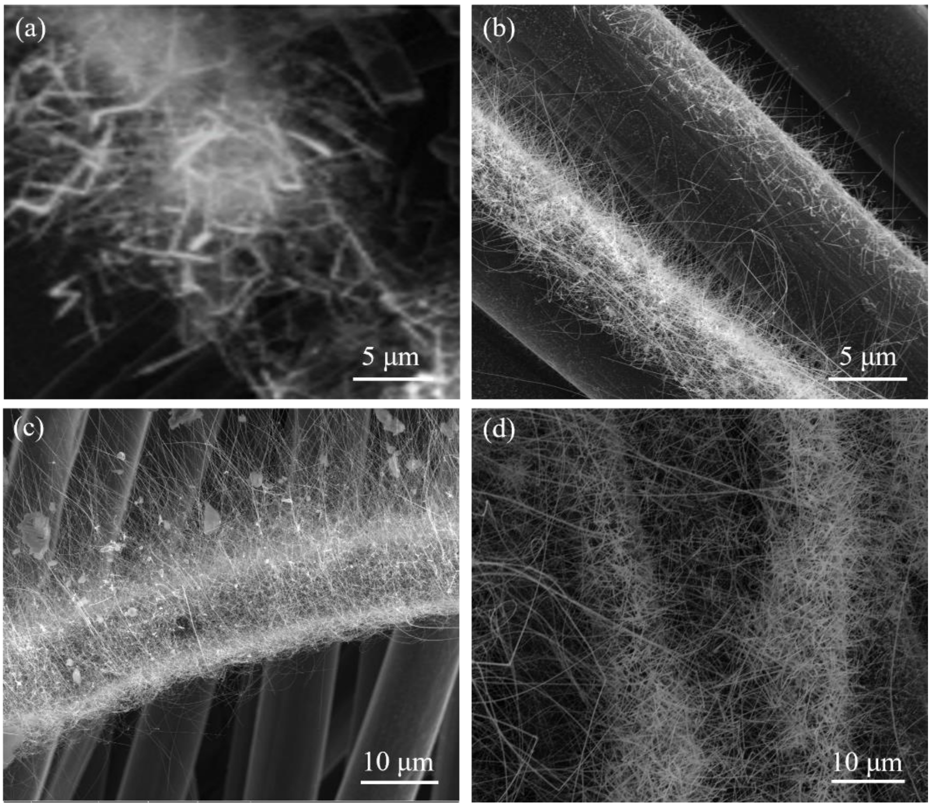


**Figure S7** SEM images of InN nanowire growth on carbon fibers with varying gold deposition times: (a) (b) 5s, (c) 10s and (d) 20s.

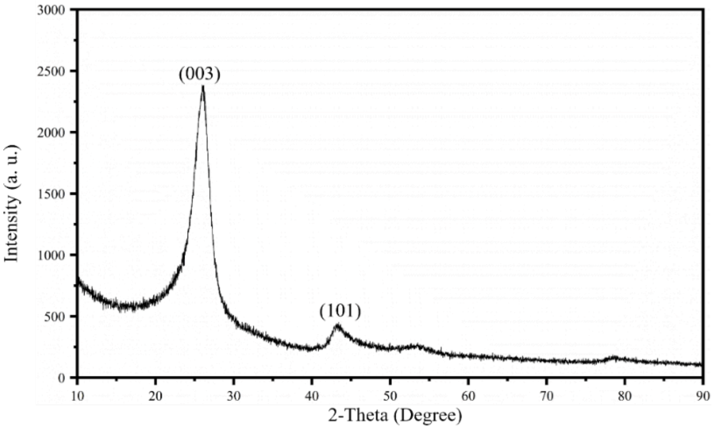


**Figure S8** XRD patterns of the pure carbon fibers.

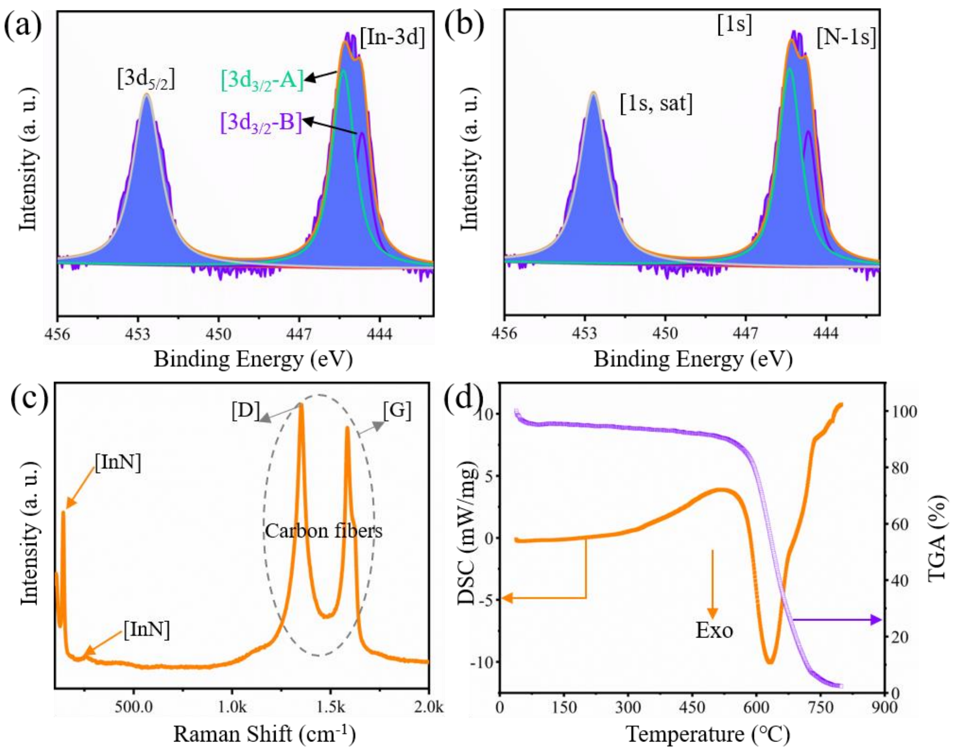


**Figure S9** XPS spectra depicting distinct peaks for (a) In3d and (b) N1s. (c) Raman spectra displaying characteristic InN adsorption peaks and the D and G bands of carbon fibers. (d) TGA-DSC curves of the InN/Au-CFs composite at a heating rate of 5 °C min$^{-1}$ in air.

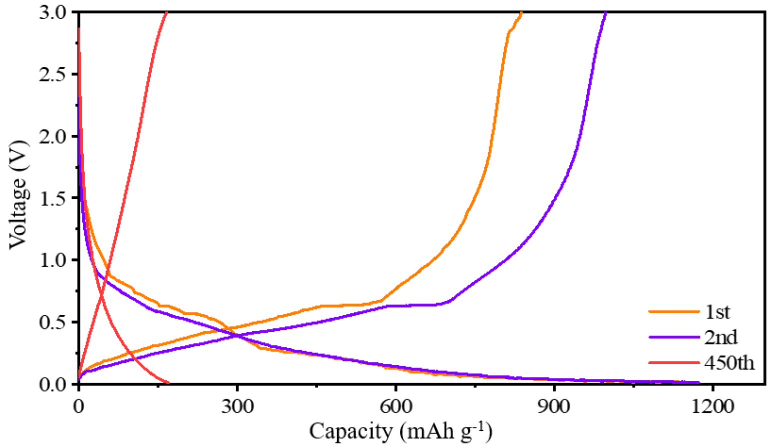


**Figure S10** The discharge/charge profiles of the InN-CFs at 0.1 A g$^{-1}$.

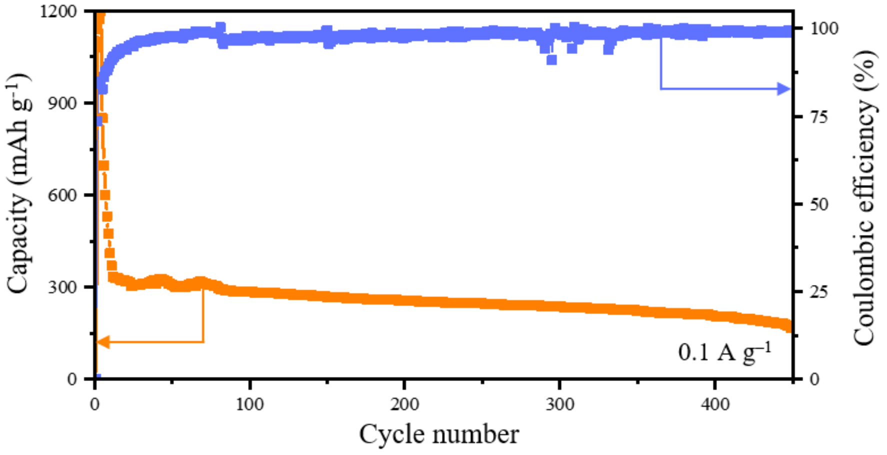


**Figure S11** The cycling performance of the InN-CFs anode at 0.1 A g$^{-1}$.

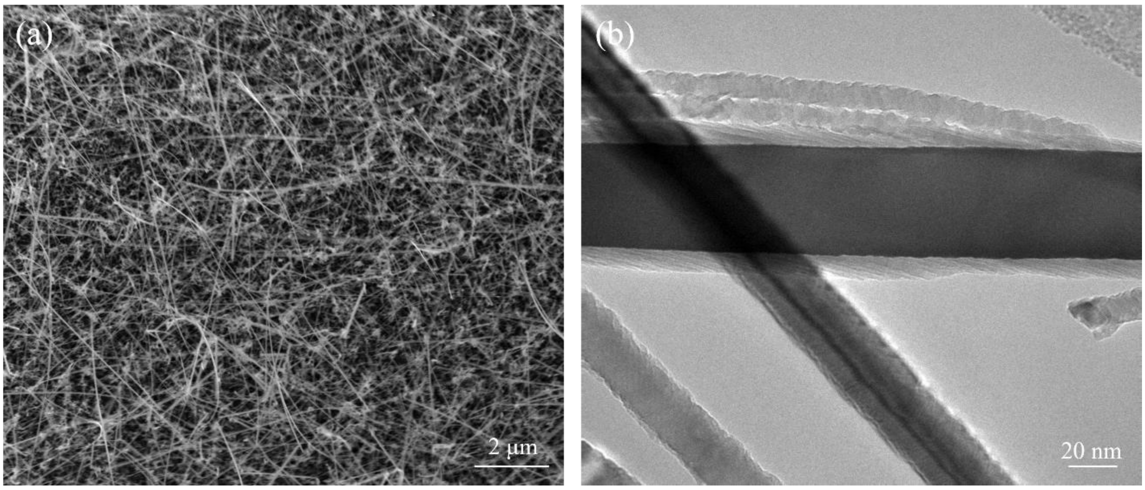


**Figure S12** SEM (a) and TEM (b) iamges of InN/Au-CFs after 450 cycles, respectively.

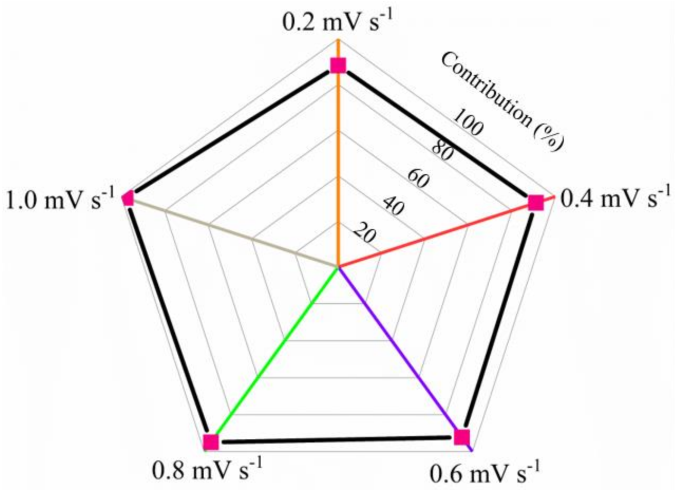


**Figure S13** Contribution ratio of pseudocapacitive and diffusion-controlled charges at various scan rates.

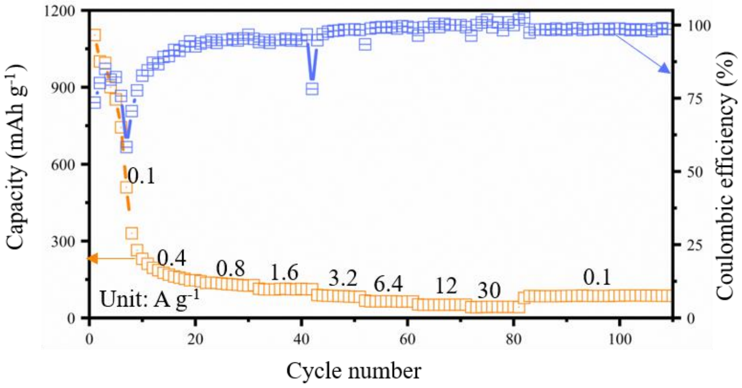


**Figure S14** The rate performance of the InN-CFs anode.

**Table S1** Comparison of the capacitance of 1D anode materials for LIBs prepared in previous works with those prepared in this work

| Sample | Current density (mA $g^{-1}$) | Cycle | Capacity (mAh $g^{-1}$) | Ref. |
|---|---|---|---|---|
| Artificial Graphite | ~ | 8000 | 350 | [1] |
| Bi/carbon nanofibers | 100 | 500 | 316.7 | [2] |
| $CoSe_2$/carbon nanofibers | 200 | 400 | 430 | [3] |
| $Bi_2O_3$/carbon nanofibers | 100 | 200 | 430 | [4] |
| $Bi_2Se_3$/carbon nanofibers | 100 | 260 | 443 | [5] |
| $Cu/Cu_2O$ nanowires | 1000 | 500 | 401 | [6] |
| $ZnO@SnO_2$ nanowires | 100 | 150 | 686 | [7] |
| $CoP@Co_2P$ nanorods | 100 | 300 | 621 | [8] |
| CoP/carbon nanowires | 200 | 200 | 640 | [9] |
| $VS_4$ nanowires | 400 | 120 | 129 | [10] |
| This work | 100 | 450 | 632.5 | This work |

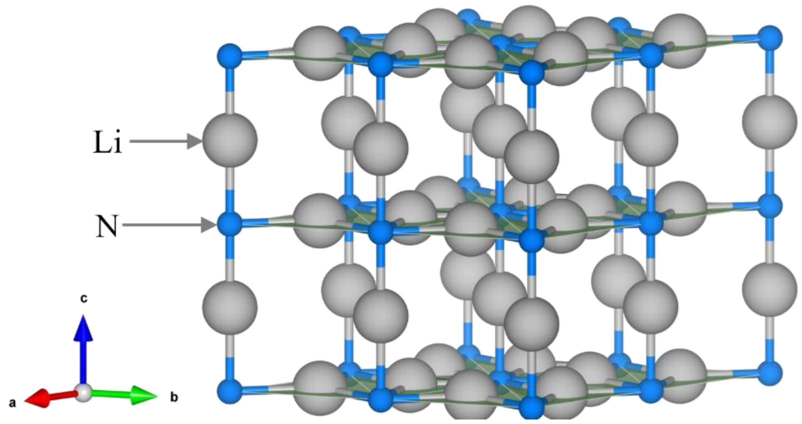


**Figure S15** The crystal structure of $Li_3N$. The blue ball represents lithium atom and the green ball represents nitrogen atom, respectively.

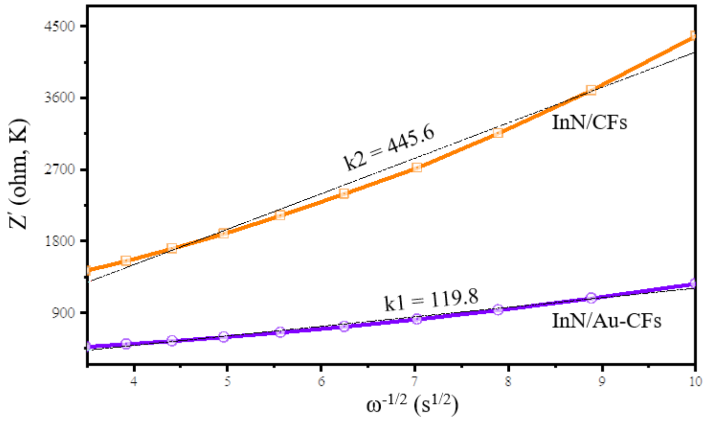


**Figure S16** The real part of the complex impedance versus $\omega^{-1/2}$ at open-circuit voltage.

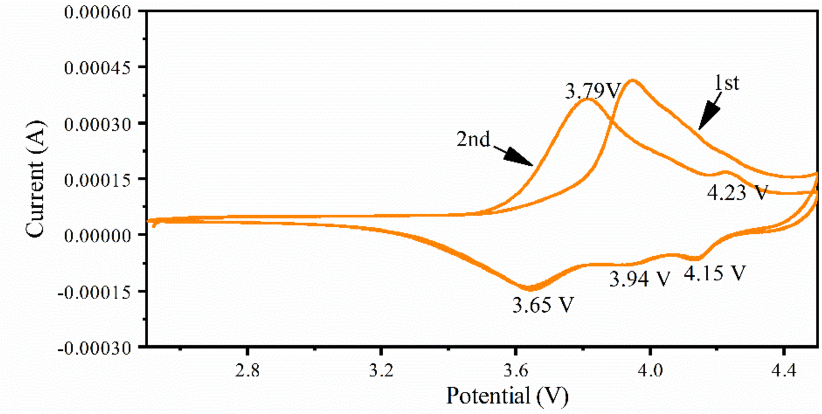


**Figure S17** The CV profiles of the Al-NCZ||InN/Au-CFs full cell with a scan rate of 0.2 mV $s^{-1}$.

**Appendix**

**Appendix 1** Description of non-Faradaic or Faradaic behavior.

With increase of the scan rate, the peak current increases without being proportional to square root of the scan rate, which illustrates that the charge and discharge processes are composed of non-Faradaic and Faradaic behavior. The relationship between peak current ($i$) and scan rate ($v$) could be illustrated by the equations as follows:

$$i = av^{b} \quad \text{(\textbf{Equation S1})}$$

$$\log(i) = b\log(v) + \log(a) \quad \text{(\textbf{Equation S2})}$$

where a and b are adjustable parameters**.** The b value determines the style of Li-ion insertion/extraction. When the value of b is 0.5, the electrochemical reaction is dominated by ionic diffusion (non-Faradaic). When the value of b is 1, the process is mainly decided by pseudocapacitive (Faradaic) control.[11]

**Appendix 2** Description of equipment circuit and calculation of ion diffusion.

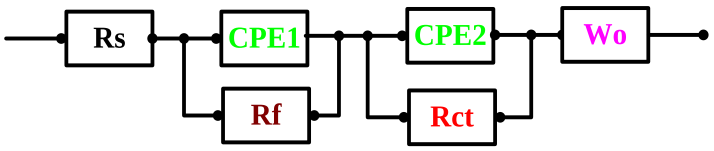


Where $R_s$ is the solution resistance of the bulk electrolyte, CPE is Constant Phase Element, $R_f$ is the Li-ion migration resistance, $R_{ct}$ is the charge transfer resistance, $W_o$-R is the Warburg Open Circuit Terminus impedance, $W_o$-T is the Open Circuit Terminus T parameter values, and $W_o$-P is the Open Circuit Terminus P parameter values.[12]

Electric conductivity and Li-ion diffusion coefficient at open circuit state can be calculated from

$$D = 2R^2T^2A^{-2}n^{-4}F^{-4}C^{-2}\sigma^{-2} \qquad \textbf{(Equation S3)}$$

$$Z_{Re} = K + \sigma\omega^{-1/2} \qquad \textbf{(Equation S4)}$$

where D is the diffusion coefficient ($cm^2\ s^{-1}$), R is the gas constant (8.31 J $mol^{-1}$ $K^{-1}$), T is the absolute temperature (298 K), A is the surface area of the anode (0.5 $cm^2$), n is the number of electrons transferred in the half-reaction for the redox couple, F is the Faraday constant (96485 C $mol^{-1}$), C is the is the molar concentration of Li-ions in In/Au-CFs electrode, K is a constant, $\omega$ is frequency, and $\sigma$ is the Warburg factor which corresponds to the slope of the curve shown in **Figure 4**g.[13]

**Appendix 3** Preparation of Al-NCZ cathodes.

*Preparation of $LiNi_{0.8}Co_{0.15}Zn_{0.05}O_2$*: $LiNi_{0.8}Co_{0.15}Zn_{0.05}O_2$ were prepared by the sol-gel method in our previous report using citric acid as a chelating agent. Stoichiometric amount of reactants $Zn(NO_3)_2{\cdot}6H_2O$ (AR, 99%), $Ni(CH_3COO)_2{\cdot}4H_2O$ (AR, 99%), $Co(CH_3COO)_2{\cdot}4H_2O$ (AR, 99%) and $CH_3COOLi{\cdot}2H_2O$ were dissolved in deionized water to give a solution with mild stirring. An aqueous solution of citric acid was added at 1:1 molar ratio with the total transition metal ions. The pH of the mixed solution was maintained at 6.7 by adding ammonium hydroxide solution. Thereafter, the mixed solution was constantly shocked at about 80 °C for 4 h. Then, the gel was dried in oven at 120 °C for 12 h, forming the amorphous powders. The resulted amorphous powder was disposed at 500 °C for 5 h in air to remove the organic contents. Then, the as-prepared precursor was ground to fine powders and calcined at 700 °C for 10 h in oxygen to obtain the final product.[14]

*Preparation of $Al_2O_3$ coated $LiNi_{0.8}Co_{0.15}Zn_{0.05}O_2$*: a typical experiment is as following: 100 mg $LiNi_{0.8}Co_{0.15}Zn_{0.05}O_2$ was dispersed in N, N-Dimethylformamide (DMF) and dispersed by ultrasonic for 60 minutes. After stirred for 3 h, 15 mg aluminium isopropoxide (AR, 99%) was added in the mixed liquor. Finally, the mixture was dried in oven at 120 °C for 12 h, and calcined at 400 °C in oxygen for 6 h to form $Al_2O_3$ coated $LiNi_{0.8}Co_{0.15}Zn_{0.05}O_2$ cathode material ($Al_2O_3$-coated $LiNi_{0.8}Co_{0.15}Zn_{0.05}O_2$ denoted as Al-NCZ).

**References**

[1] Y. Liu, H. Shi, Z.-S. Wu, *Energ. Environ. SCi.* **2023**, 16, 4834.

[2] H. Yin, Q. Li, M. Cao, W. Zhang, H. Zhao, C. Li, K. Huo, M. Zhu, *Nano Res.* **2017**, 10, 2156.

[3] H. Yin, H.-Q. Qu, Z. Liu, R.-Z. Jiang, C. Li, M.-Q. Zhu, *Nano Energy* **2019**, 58, 715.

[4] H. Yin, M.-L. Cao, X.-X. Yu, H. Zhao, Y. Shen, C. Li, M.-Q. Zhu, *Mater. Chem. Front.* **2017**, 1, 1615.

[5] H.-Q. Qu, H. Yin, Y.-L. Wang, C. Fan, K. N. Hui, C. Li, M.-Q. Zhu, *Mater. Chem. Front.* **2021**, 5, 2832.

[6] Y. Wang, L. Cao, J. Li, L. Kou, J. Huang, Y. Feng, S. Chen, *Chem. Eng. J* **2020**, 391, 123597.
[7] J. Yan, P. Xu, S. Chen, G. Wang, F. Zhang, W. Zhao, Z. Zhang, Z. Deng, M. Xu, J. Yun, Y. Zhang, *Electrochim. Acta* **2020**, 330, 135312.
[8] F.-F. Li, J.-F. Gao, Z.-H. He, L.-B. Kong, *ACS Appl. Mater. Interfaces* **2021**, 13, 10071.
[9] H. Li, Y. Zhu, K. Zhao, Q. Fu, K. Wang, Y. Wang, N. Wang, X. Lv, H. Jiang, L. Chen, *J Colloid Inerf Sci* **2020**, 565, 503.
[10] L. Xing, K. A. Owusu, X. Liu, J. Meng, K. Wang, Q. An, L. Mai, *Nano Energy* **2021**, 79, 105384.
[11] N. Wu, P. H. Chien, Y. T. Li, A. Dolocan, H. H. Xu, B. Y. Xu, N. S. Grundish, H. B. Jin, Y. Y. Hu, J. B. Goodenough, *J. Am. Chem. Soc.* **2020**, 142, 2497.
[12] Y. Yuan, Z. W. Chen, H. X. Yu, X. K. Zhang, T. T. Liu, M. T. Xia, R. T. Zheng, M. Shui, J. Shu, *Energy Storage Mater.* **2020**, 32, 65.
[13] Y. J. Cheng, Y. Y. Dou, D. X. Kan, Y. Z. Wang, Y. J. Wei, *Appl. Surf. Sci.* **2023**, 610, 155481.
[14] H. Yin, X.-X. Yu, H. Zhao, C. Li, M.-Q. Zhu, *J. Solid State Electr.* **2018**, 22, 2395.
